# Role of protein and mRNA oxidation in seed dormancy and germination

**DOI:** 10.3389/fpls.2013.00077

**Published:** 2013-04-08

**Authors:** Hayat El-Maarouf-Bouteau, Patrice Meimoun, Claudette Job, Dominique Job, Christophe Bailly

**Affiliations:** ^1^UR5 EAC7180 CNRS, Université Pierre et Marie Curie Paris 06Paris, France; ^2^CNRS/Université Claude Bernard Lyon 1/Bayer CropScience Joint laboratory (UMR 5240)Lyon, France

**Keywords:** dormancy, germination, mRNA, oxidation, proteins, reactive oxygen species, seed

## Abstract

Reactive oxygen species (ROS) are key players in the regulation of seed germination and dormancy. Although their regulated accumulation is a prerequisite for germination, the cellular basis of their action remains unknown, but very challenging to elucidate due to the lack of specificity of these compounds that can potentially react with all biomolecules. Among these, nucleic acids and proteins are very prone to oxidative damage. RNA is highly sensitive to oxidation because of its single-stranded structure and the absence of a repair system. Oxidation of mRNAs induces their decay through processing bodies or results in the synthesis of aberrant proteins through altered translation. Depending on the oxidized amino acid, ROS damage of proteins can be irreversible (i.e., carbonylation) thus triggering the degradation of the oxidized proteins by the cytosolic 20S proteasome or can be reversed through the action of thioredoxins, peroxiredoxins, or glutaredoxins (cysteine oxidation) or by methionine sulfoxide reductase (methionine oxidation). Seed dormancy alleviation in the dry state, referred to as after-ripening, requires both selective mRNA oxidation and protein carbonylation. Similarly, seed imbibition of non-dormant seeds is associated with targeted oxidation of a subset of proteins. Altogether, these specific features testify that such oxidative modifications play important role in commitment of the cellular functioning toward germination completion.

## INTRODUCTION

The chemical energy sequestered in plants during photosynthesis accumulates in seeds, which are the major genetic delivery systems essential for plant biodiversity but also provide a food source for animals and humans. Most seeds of plants growing in temperate climates are dispersed in a dry mature state (called orthodox seeds). They will pass through the complex process of germination provided they are non-dormant and if the environmental conditions are favorable, and the result is a young growing plant (i.e., a seedling) from a quiescent dry seed. Seed germination commences with the uptake of water and is completed with the appearance of the embryo through the seed surrounding structure(s). It is tightly regulated by temperature, oxygen, and light conditions. Germination is also controlled by endogenous factors such as the plant hormones abscisic acid (ABA) and gibberellins (GA) that play a major role in regulating early seed germination through the process of dormancy, which is a block to the completion of germination of a mature intact viable seed (Finch-Savage and Leubner-Metzger, 2006). This evolutionary trait allows plant species to survive through unfavorable seasons and enables seeds to remain quiescent until the conditions for germination and seedling establishment become favorable again (Finch-Savage and Leubner-Metzger, 2006; Donohue et al., 2010). Therefore dormancy alleviation is associated with a widening of the environmental conditions allowing seed germination.

Despite the huge progress that has been made the last decade owing to the emergence of the -omics approaches, the molecular mechanisms regulating seed germination and dormancy are far from being resolved. For example, how a dormant seed acquires the ability to germinate during a period of dry storage after harvest, so called after-ripening, is unknown. Reactive oxygen species (ROS) have been proposed to be key players in seed germination and dormancy (Bailly, 2004; Oracz et al., 2007; Bailly et al., 2008; El-Maarouf-Bouteau and Bailly, 2008). It appears that seed germination occurs when the seed ROS content is enclosed within an oxidative window that allows ROS signaling but not ROS damage (Bailly et al., 2008). ROS are short-lived and reactive compounds so that their effect as signaling molecules may be mediated by secondary messengers, such as proteins or even nucleic acids (Møller and Sweetlove, 2010). The purpose of this review is to show that the beneficial effect of ROS drives cell functioning toward germination by specific oxidation of proteins and mRNAs.

## SEEDS CONTINUOUSLY PRODUCE ROS

Reactive oxygen species originate from the reduction of oxygen, which mainly gives rise to the most common species superoxide ($O_{2}^{-\cdot }$), hydrogen peroxide (H_2_O_2_), and hydroxyl radical (HO^·^). Detailed mechanisms of ROS production in seeds have already been described (Bailly, 2004), but it has to be underlined that in a same organism, i.e., a seed, ROS can be produced by non-enzymatic or enzymatic processes, depending on the developmental stage of the seed and on its moisture content. When seeds are stored dry after their final maturation on the mother plant, ROS are issued from autoxidative reactions that occur spontaneously and continuously. In contrast, seed imbibition is associated with a tight regulation of ROS homeostasis, which involves both ROS producing enzymes, such as nicotinamide adenine dinucleotide phosphate (NADPH) oxidases, and ROS detoxifying enzymes (catalase, superoxide dismutase,…). Attention has often been paid to identifying ROS sensors and transduction networks transmitting information about their homeostasis within cell compartments or even from cell to cell (Mittler et al., 2011). H_2_O_2_ is widely recognized for acting as a cellular messenger because of its relative stability and because it can cross membranes (Møller et al., 2007). However, H_2_O_2_ is a simple molecule and it does not have the required specificity to selectively trigger complex cellular processes, as those that are involved in the control of seed germination. This implies that H_2_O_2_ is likely to act as a primary messenger by oxidizing compounds that will in turn act as second messengers (Møller and Sweetlove, 2010).

## OXIDATION OF MESSENGER RNAs REGULATES SEED TRANSLATIONAL ACTIVITY

Seeds accumulate high amounts of mRNAs during their maturation program. It has been demonstrated that the pool of mRNA accumulated during seed development is used upon imbibition to ensure translation of the proteins required for completing germination (Rajjou et al., 2004; Nakabayashi et al., 2005; Kimura and Nambara, 2010). As a consequence, *de novo* transcription is probably not essential during early stages of germination (Rajjou et al., 2004), thus suggesting that the pool of stored mRNA that is translated during the early steps of seed imbibition governs dormancy expression and germination potential. This implies that the pool of transcripts translated during seed imbibition differs between non-dormant and dormant seeds. Selective oxidation of a pool of transcripts during after-ripening or early seed imbibition is an attractive mechanism for explaining rapid modifications of the pattern of translated proteins. RNA is much more vulnerable to oxidative damage than DNA because of its single-stranded structure, its cytoplasmic location and the absence of efficient repair systems to minimize oxidative damage (Kong and Lin, 2010). Many oxidative damages of bases have been identified in RNA (Barciszewski et al., 1999) but the most common oxidized base in RNA is guanine, from which oxidation produces 8-hydroxyguanosine (8-OHG). 8-OHG results from a reaction of a HO· with guanine to form a 3-hydroxyoctanoylcarnitine (C8-OH) adduct radical and subsequently 8-OHG after the loss of an electron and proton. 8-OHG can be detected and quantified in tissues using high-performance liquid chromatography-electrochemical detection (HPLC-ECD) or antibodies. To date, mRNA oxidation has mainly been observed in a wide range of diseases in animals and humans (Poulsen et al., 2012). In Alzheimer’s disease, for example, up to 50% of mRNAs in frontal cortices are oxidized (Shan and Lin, 2006). In a pioneering study, Bazin et al. (2011) recently reported that mRNA oxidation occurs during sunflower seed dry after-ripening. They showed that the amount of 8-OHG in poly(A)-RNA increases by 50% during dormancy alleviation in the dry state, whilst this increase was not detectable when using the total RNA population, which indicates that mRNAs were more sensitive to oxidation than other RNA species in this system. As in animals systems (Kong and Lin, 2010), this mRNA oxidation in seeds was not random but highly selective, mainly targeting 24 stored mRNAs during sunflower seed after-ripening, most of them corresponding to genes involved in cell signaling (Bazin et al., 2011). For example, among these oxidized transcripts, protein phosphatase 2C PPH1, mitogen-activated protein kinase phosphatase 1, and phenyl ammonia lyase 1 were identified. However, the molecular basis for such selectivity is so far not known. Nevertheless, selective oxidation of transcripts is highly relevant in the context of dormancy and germination when one considers the consequence of this process. Although repair of oxidized RNAs has sometimes been reported (Aas et al., 2003), the major issues of oxidized mRNAs are the suppression of the protein synthesis and their degradation. As non-oxidized mRNAs, oxidized mRNAs are recognized by ribosomes (Tanaka et al., 2007), but the presence of oxidized bases causes translation errors and produces truncated proteins mainly because of premature termination (Tanaka et al., 2007; Chang et al., 2008). Bazin et al. (2011) and Chang et al. (2008) demonstrated that mRNA oxidation caused reduced protein expression. In addition, it has been suggested that P-bodies, which include decapping enzymes, activators of decapping, and 5′–3′ exonuclease, specifically degrade oxidized mRNAs and clean the cell from these compounds (Sheth and Parker, 2006; Moreira et al., 2008). Interestingly, it was demonstrated that ROS induced the formation of cytoplasmic P-bodies (Shan et al., 2007). The work of Bazin et al. (2011) therefore highlights a potential mechanism of seed dormancy alleviation in which targeted mRNA oxidation can fine-tune the cell signaling pathway that controls germination by targeting mRNA decay and by regulating selective translation. It is of outstanding interest to determine whether this process, which can rapidly regulate cell functioning, is involved in other plant developmental processes or in response to stresses.

## SELECTIVE PROTEIN OXIDATION DRIVES THE GERMINATION PROCESS

Proteins are, with nucleic acids and lipids, the most sensitive molecules to *in vivo* oxidation. Protein oxidation can be caused directly by ROS or by co-products of oxidatively modified lipids, amino acids, or sugars (Shacter, 2000). There are numerous different types of protein oxidative modifications. Oxidative attack of amino acyl moieties, such as Lys, Arg, Pro, and Thr, induces formation of carbonyl groups (aldehydes and ketones) on the side chains (Nyström, 2005). Carbonylation is irreversible and not repairable (Dalle-Donne et al., 2003). To avoid their toxic accumulation, carbonylated proteins are degraded through the action of the 20S proteasome in the cytosol (Nyström, 2005).

Besides carbonylation, the two amino acids that are the most prone to oxidative attack are Cys and Met, both of which contain susceptible sulfur atoms (Shacter, 2000). Oxidation of Cys by ROS promotes reversible disulfide bond formation and can also generate the oxidized derivatives sulfenic acid (Cys SOH), sulfinic acid (Cys SO_2_H), or sulfonic acid (Cys SO_3_H; Rinalducci et al., 2008). The major products of Met oxidation are *S*- and *R*-diastereomers of Met sulfoxide (MetSO), which can be reversed through the specific action of methionine sulfoxide reductase (MSR) A and B on *S*- and *R*-diastereomers, respectively (Davies, 2005). In addition, a more severe attack of Met can result in the irreversible formation of sulfone (MetSO_2_).

Job et al. (2005) demonstrated that massive protein oxidation occured during *Arabidopsis* seed germination. It was shown that 12S subunits of cruciferin, a legumin-type reserve protein, became oxidized during seed maturation and that this protein disappeared steadily upon seed imbibition reflecting its mobilization during plantlet establishment. During pea seed germination, Barba-Espín et al. (2011) also observed carbonylation of protein reserves such as vicilins and pea albumin 2. Carbonylation of seed storage proteins would help trigger their mobilization during germination by destabilizing the compact seed storage protein complexes, thus increasing their susceptibility toward proteolytic cleavage by 20S proteasome (Job et al., 2005). Also, because of their abundance, these seed storage proteins might be viewed as an efficient scavenging system for ROS that are actively generated during germination. In parallel, the carbonylation of many proteins from the albumin fraction occurred during imbibition of *Arabidopsis* and pea seeds (Job et al., 2005; Barba-Espín et al., 2011). Oxidation was not randomly distributed but targeted specific proteins such as glycolytic enzymes, mitochondrial ATP synthase, aldose reductase, methionine synthase, translation factors, and several molecular chaperones (Job et al., 2005). Despite such accumulation of carbonylated proteins, usually viewed as a factor of deterioration in the context of aging in a variety of model systems, *Arabidopsis* seeds germinated at a high rate and yielded vigorous plantlets suggesting that this oxidative process is not deleterious. Specific protein carbonylation might be required for protecting other cell components against the effects of ROS that are issued from the recovery of metabolic activity during seed imbibition. It can also be hypothesized that impairment of some metabolic activities such as the oxidation of glycolytic enzymes, for example, could lead to the activation of the pentose phosphate pathway (PPP) thus providing reducing power to the germinating seed (Job et al., 2005; Barba-Espín et al., 2011). This is in good agreement with previous work suggesting a key role for PPP in the completion of seed germination (Bewley and Black, 1994).

Studying the relationship between dormancy and protein carbonylation brought several other lines of evidence showing the beneficial effect of carbonylation in seed germination. Thus, Oracz et al. (2007) documented that sunflower seed dry after-ripening is associated with ROS accumulation, which induces selective protein oxidation in anhydrobiosis. Such a process appears to be a prerequisite for dormancy alleviation since preventing ROS production by storing dormant seeds at low moisture content blocks this process. This mechanism has to be considered with regards to the recent findings of Bazin et al. (2011) since both studies clearly document that non-enzymatic processes leading to selective translation and selective protein degradation are probably key events occurring during after-ripening. Müller et al. (2009) also showed that *Arabidopsis* seed after-ripening is associated with increased protein carbonylation. In addition, carbonylation of specific proteins occurs upon treating dormant sunflower seeds with either hydrogen cyanide or methylviologen, two dormancy-releasing compounds (Oracz et al., 2007). Oracz et al. (2007) have discussed the role of protein oxidation in dormancy alleviation with regards to the nature of the carbonylated proteins identified. For example these authors showed that alcohol dehydrogenase is carbonylated in axes of all seeds undergoing germination, which fits very well with the beneficial effect of alcohol on breaking seed dormancy (Cohn et al., 1989; Corbineau et al., 1991). As in *Arabidopsis* (Job et al., 2005), the oxidation of seed reserve proteins seems also to be associated with sunflower seed germination (Oracz et al., 2007).

Reversible disulfide bond formation from thiol groups of cysteine is probably the post-translational oxidative modification that has been the most extensively studied in seed physiology (Buchanan and Balmer, 2005; Arc et al., 2011). On the one hand, oxidative conditions occurring during seed germination (Bailly, 2004) are likely to promote formation of disulfides bonds and of mixed disulfides between glutathione and cysteinyl residues (i.e., glutathionylation; Buchanan and Balmer, 2005). Formation of disulfide bridges and glutathionylation would prevent irreversible oxidation of proteins and their subsequent degradation (Dalle-Donne et al., 2007; Spadaro et al., 2010). In addition, disulfide bridges alter protein folding, stability and potential activity (Buchanan and Balmer, 2005). For example, some components of translation and transcription machineries are regulated by the redox status and by ROS homeostasis (Buchanan and Balmer, 2005), but the redox regulation of transcription and translation in germinating seeds has not been demonstrated yet. Interestingly, Bykova et al. (2011) recently showed that wheat seed dormancy is regulated by the protein redox thiol status during imbibition. The redox changes occurring during imbibition of non-dormant seeds, relating to ROS accumulation, would be associated with a dramatic redox change of protein thiols, thus driving cell functioning to germination, probably by interacting with hormone signaling pathways (Bykova et al., 2011). On the other hand, seed germination is associated with the reduction of disulfide bonds owing to the regulatory action of thioredoxins, peroxiredoxins and glutaredoxins (e.g., Marx et al., 2003; Wong et al., 2004; Alkhalfioui et al., 2007). Formation of disulfide bonds occurs during maturation drying and this process would be associated with the down regulation of metabolic activities in mature seed (Buchanan and Balmer, 2005). In contrast, the resumption of an active metabolism during seed imbibition would be related to the reduction of disulfide bonds of metabolic proteins. It has also been proposed that the mobilization of protein reserves during germination would require disulfide bond reduction since it increases solubility and facilitates proteolytic attacks (Alkhalfioui et al., 2007). In summary, the studies related to the redox regulation of thiol groups during germination bring a contrasted picture of their putative role. This suggests that germination might be associated with a subtle balance between oxidation and reduction of cysteines on targeted proteins. Attention will have to be paid in the forthcoming years to better elucidate the roles of thiol oxidation in seed germination. One cannot exclude that the selective oxidation of thiols by ROS could act in concert with the reduction of disulfides by thioredoxin, peroxiredoxins, and glutaredoxins.

The consequences of Met redox changes are well documented in mammals. Oien and Moskovitz (2008) proposed that the enzymatic regeneration of MetSO through MSR activity falls into three categories: regulation of signaling pathways, Met acting as an antioxidant for protecting proteins from higher oxidative events and damage to protein function and subsequent formation of protein-carbonyl adducts (Moskovitz and Oien, 2010). The MSR system is present in most organisms from bacteria to human and it has been well described by Rouhier et al. (2006) and Tarrago et al. (2009) in plants. However, little information is available on MSR substrates, i.e., oxidized methionine in proteins, due to the difficulty to isolate these oxidized targets. Recently, a method allowing MetSO identification by COFRADIC (combined fractional diagonal chromatography) proteomics technology has been set up in mouse serum proteome (Ghesquiere et al., 2011). This permitted the identification of 35 *in vivo* oxidized methionine sites in 27 different proteins. In leaf extracts, Tarrago et al. (2012) identified 24 protein partners of *Arabidopsis* plastidial MSRB1 using a strategy based on affinity chromatography.

The major challenge of the upcoming studies dealing with protein oxidation in seeds will certainly be to build up a comprehensive scheme integrating the relationship between the various post-translational modifications of proteins. For example, the relationship between carbonylation, Met sulfoxidation and phosphorylation have been unraveled recently showing that sulfoxidation alters protein carbonylation and phosphorylation (Hardin et al., 2009; Moskovitz and Oien, 2010; Oien et al., 2011).

## CONCLUDING REMARKS

**FIGURE 1:**
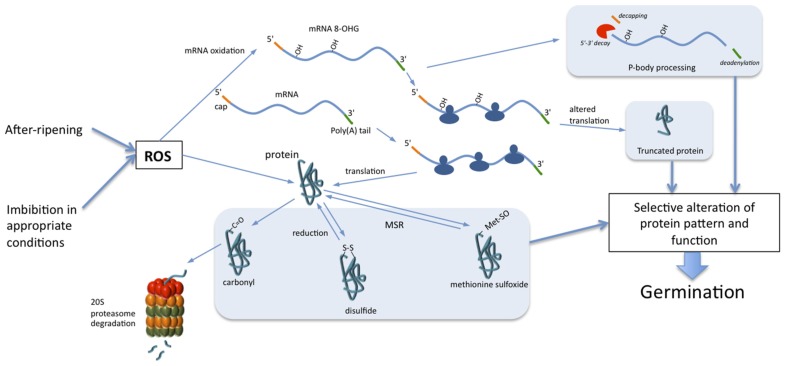
**Role of mRNA and protein oxidation in seed germination.** During non-dormant seed imbibition, ROS, accumulated during dry after-ripening or produced during imbibition, trigger selective oxidation of mRNA and proteins. Oxidation of mRNAs leads to their decay through the action of P-bodies and/or to the alteration of translation. Proteins can be irreversibly oxidized by carbonylation, which leads to their cytosolic degradation through the 20S proteasome. Oxidation of cysteine and methionine induces formation of disulfide bonds and methionine sulfoxide, respectively. Reduction of disulfide bonds occurs through the action of thioredoxins, peroxiredoxins, or glutaredoxins when methionine sulfoxides are reduced to methionine by methionine sulfoxide reductase (MSR). The selective oxidation of a subset of mRNAs and proteins guides cell signaling pathways toward the completion of seed germination.

The discovery of the occurrence of oxidative modifications of proteins and mRNAs in seed germination and breaking of dormancy opens new avenues to better understand such complex developmental processes. The identification of mRNA oxidation in seeds allows proposing a hypothesis for explaining the mechanism underlying selective translation in non-dormant seeds. In addition, the selective oxidative post-translational modifications of proteins claim for a strong involvement of the suppression of negative regulators of germination in the mechanisms associated with dormancy release (**Figure 1**). These findings allow proposing the hypothesis that ROS accumulation during seed imbibition and subsequent oxidation of targeted mRNAs and proteins are important features for regulation of seed germination, which will be the topic of future studies.

## Conflict of Interest Statement

The authors declare that the research was conducted in the absence of any commercial or financial relationships that could be construed as a potential conflict of interest.
